# Numerical Simulation of Supersonic Gap Flow

**DOI:** 10.1371/journal.pone.0117012

**Published:** 2015-01-30

**Authors:** Xu Jing, Huang Haiming, Huang Guo, Mo Song

**Affiliations:** Institute of Engineering Mechanics, Beijing Jiaotong University, Beijing, 100044, China; University of Florida, UNITED STATES

## Abstract

Various gaps in the surface of the supersonic aircraft have a significant effect on airflows. In order to predict the effects of attack angle, Mach number and width-to-depth ratio of gap on the local aerodynamic heating environment of supersonic flow, two-dimensional compressible Navier-Stokes equations are solved by the finite volume method, where convective flux of space term adopts the Roe format, and discretization of time term is achieved by 5-step Runge-Kutta algorithm. The numerical results reveal that the heat flux ratio is U-shaped distribution on the gap wall and maximum at the windward corner of the gap. The heat flux ratio decreases as the gap depth and Mach number increase, however, it increases as the attack angle increases. In addition, it is important to find that chamfer in the windward corner can effectively reduce gap effect coefficient. The study will be helpful for the design of the thermal protection system in reentry vehicles.

## Introduction

Thermal protection system (TPS) performance is critical, since mass reduction trades directly with increase in the science payload for a given reentry mass or reduction in launch vehicle cost by using a lighter entry system and a smaller launch vehicle [1]. As is known to all, numerous gaps exist in TPS of the supersonic aircraft. For example, TPS of the space shuttle consists of various ceramic insulation tiles. To avoid extrusion damage of ceramic tiles suffering from the heat-expansion and cold-contraction, gaps must be reserved between these tiles. These gaps are able to disrupt the flow field and increase the local heat flux, which could damage the heat shield, and the heat radiation effect in the narrow gaps is blocked, so low heat flux may lead to local overheating. Therefore it is necessary to study the local thermal environment around the gaps under different flight conditions.

Aerodynamic heating flow field around the gap is complex. In recent years, there has been increasing research interesting in the experiment and simulation on the flow field around the gap. A number of previous investigators have considered that empirical and semi-empirical formulas are developed according to experimental data which come from ground and flight tests. For example, Tang [2] from the institute of mechanics Chinese academy of science conducted wind tunnel tests of free-stream at Mach numbers of 9.85,12 and 15.5, where heat transfer distributions in a gap were measured with a sharp leading edge flat plate and a flat cylinder, and effects of Mach number, attack angle and deflection on heat transfer to the gap wall were discussed. Mori [3] proposed an optical measurement technique for the heat flow onto ‘Shaded’ area in the hypersonic flows, in which the present method employs temperature sensitive paint and simple optics installed inside of a model, but the precision of it is still open to dispute. Furthermore, in order to redisplay the flow characteristic of high-temperature gas, relatively few studies have considered the simulation on compressible flow. Huang *et al* [4] applied the vortex method to analyze the supersonic flow. Jackson [5] presented a combined experimental and computational study of laminar cavity flows at hypersonic speeds. Larcheveque *et al* [6] performed large-eddy simulation of the flow over a deep cavity, in which the change of surface pressure, the speed and the noise with time was attained. Hinderks *et al* [7] investigated hypersonic flow with consideration of fluid structure interaction, where the flow field was supposed as laminar flow, and chemical non-equilibrium conditions were adopted in the calculation. Paolicchi *et al* [8] used the direct simulation Monte Carlo method to do numerical simulation of two-dimensional steady-state hypersonic rarefied flow in a gap at different width-to-depth ratios and wall temperatures. Yang *et al* [9] developed a hypersonic aero-thermal simulation method for missile flight. Shen et al [10] studied a program of approximate numerical simulation of semi-decomposed fluid and solid coupling, and simulated the progress of a high speed airflow impacting the seal structure. However, the data are still scarce, and we need to provide more records, so further numerical simulations on the flow around a gap are still essential work at present.

In this study, two-dimensional compressible Navier-Stokes equations are solved by the finite volume method to obtain the local aerodynamic heating environment around a gap.

## Models

### Physical Models

**Fig 1:**
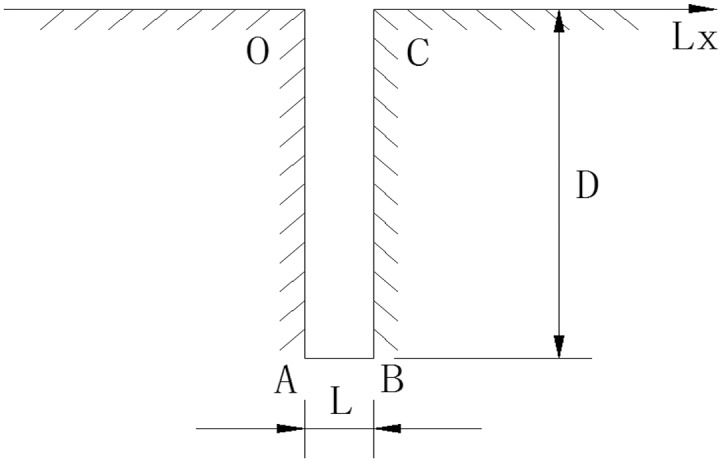
Gap.

**Fig 2:**
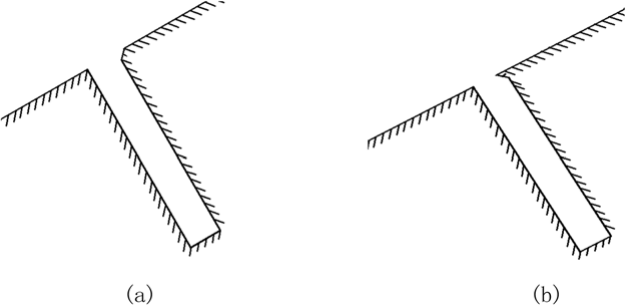
Gaps with chamfer or convex angle. (a)chamfer, (b)convex angle.

As can be seen in Fig. 1, Lx is the coordinate along the wall and its positive direction is from O to A to B to C. Firstly, when width (L)-to-depth (D) ratio L/D = 1/6, numerical models under different conditions—attack angle (*α*) = 0°,15° and 30°—are performed separately to analyze the influence of attack angle on the flow around a gap. Secondly, we hope to know the influence of width-to-depth ratio on gap effect, so models of L/D = 1/2 and 1/4 are built while width L remains 2 mm. Thirdly, chamfer and convex angles are seen in Fig. 2(a) and (b) in order to analyze the influence of chamfer and convex angle when *α* = 30° and L/D = 1/6. The length of the chamfer in Fig. 2(a) in slope direction x = 0.5mm and the length along the gap windward side y = 0.5mm. In Fig. 2(b), the length of the convex angle in slope direction x = 0.5mm and the length along the gap windward side y = 0.5mm.

**Fig 3:**
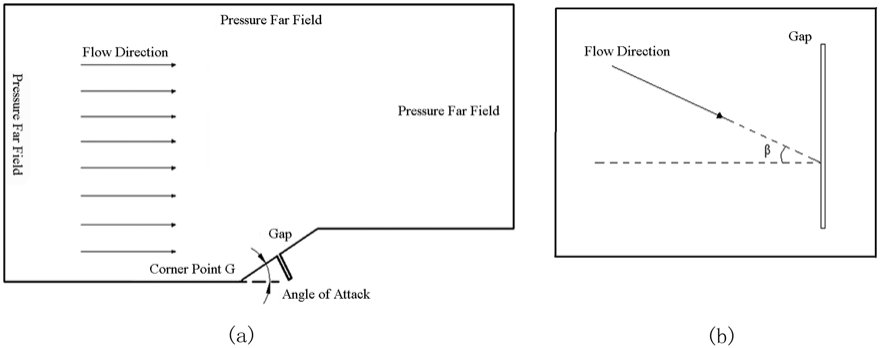
Relation between a flow and a gap. (a) Physical model, (b) Vertical view of a gap.

In Fig. 3(a), in order to match far field boundary condition, the distance between corner point G and left pressure far field boundary is 120mm, so does the distance between point G and top pressure far field boundary, while that between point G and right pressure far field boundary is 140mm. The projection of the slope in the horizontal direction is 40mm long. The various gaps above may be located in the middle of the slope. In addition, in order to study the effect of different parameter on gap local aerodynamic heating environment, smooth plate models without gaps are also built and the other sizes are as same as that in Fig. 3(a).

Define deflection angle *β* as the angle between the flow direction and the vertical direction of gaps in Fig. 3(b). If the deflection angle *β* is not equal to zero, the three-dimensional effects should be considered. But in this paper, the simulation needs to be done under the condition that the deflection angle *β* is equal to zero.

### Mathematical Model

Based on the physical model above, the two-dimensional mathematical model can be established. Governing equations for the turbulent flow around a gap are described by the differential equations

$$\frac{\partial \rho }{\partial t}+\frac{\partial }{\partial x_{i}}(\rho u_{i})=0$$ (1)

$$\frac{\partial }{\partial t}(\rho u_{i})+\frac{\partial }{\partial x_{j}}(\rho u_{i}u_{j})=-\frac{\partial P}{\partial x_{i}}+\frac{\partial }{\partial x_{j}}(\mu \frac{\partial u_{i}}{\partial x_{j}}-\rho \bar{{u^{\prime }}_{i}{u^{\prime }}_{j}})$$ (2)

$$\frac{\partial }{\partial t}(\rho T)+\frac{\partial }{\partial x_{j}}(\rho u_{j}T)=\frac{\partial }{\partial x_{j}}(\frac{k}{c}\frac{\partial T}{\partial x_{j}}-\rho \bar{{u^{\prime }}_{i}T^{\prime }})$$ (3)

Where *u*_1_, *ρ*, *P*, *T*, *µ*, *k*, *c* are the velocity component, density, pressure, temperature, viscosity coefficient, thermal conduct coefficient and specific heat capacity of fluid, respectively; *i* = 1,2; j = 1,2; Roe discrete format is adopted in the discretization of the convection term, and 5-step Runge-Kutta algorithm is used in discretization of time term.

In the above equations, S-A turbulence model is adopted on the base of Boussinesq hypothesis [11], in which turbulence variable $\bar{\mu }$ is introduced, so turbulent viscosity *µ_t_* is given in the form

$$\mu_{t}=\bar{\mu }f_{v1}$$ (4)

where ƒ_v1_ = *X^3^*/(*X^3^*+*C_v1_*), $\chi =\bar{\mu }/\mu$, and turbulence variable $\bar{\mu }$ can be deduced as

$$\frac{\partial \bar{\mu }}{\partial t}+\frac{\partial (\bar{\mu }u_{i})}{\partial x_{j}}=\left\{\nabla \left[(\mu +\bar{\mu }+C_{b2}\bar{\mu })\nabla \bar{\mu }\right]-C_{b2}\bar{\mu }\Delta \bar{\mu }\right\}/\sigma +Q$$ (5)

where *σ* and *C_b2_* are constants，*Q* is the source term in the form

$$Q=C_{b1}\bar{S}\bar{\mu }-C_{w1}f_{w}{\left(\bar{\mu }/d\right)}^{2}$$ (6)

$$\bar{S}\equiv Sf_{v3}+\bar{\mu }f_{v2}/(k^{2}d^{2})$$ (7)

$$S\equiv \sqrt{2S_{ij}S_{ij}}$$ (8)

$$S_{ij}=(\frac{\partial u_{j}}{\partial x_{i}}-\frac{\partial u_{i}}{\partial x_{j}})/2$$ (9)

$$f_{v2}={\left(1+\chi /C_{v2}\right)}^{-3}$$ (10)

$$f_{v3}=(1+\chi f_{v1})(1-f_{v2})/\chi$$ (11)

$$f_{w}=g{\left[\left(1+C_{w3}^{6})/(g^{6}+C_{w2}^{6}\right)\right]}^{1/6}$$ (12)

$$g=r+C_{w2}(r^{6}-r);r=\bar{\mu }/(\bar{S}k^{2}d^{2})$$ (13)

$$C_{w1}=C_{b1}/k^{2}+(1+C_{b2})/\sigma$$ (14)

where *d* is the distance to the wall surface, others are seen in Table 1.

**Table 1:** Model Parameters.

**C_w2_**	**C_w3_**	**C_v1_**	**C_v2_**	**C_b1_**	**C_b2_**	**k**	**σ**
0.3	2	7.1	5	0.1355	0.622	0.41	2/3

In addition, the perfect gas model is adopted in this paper, so the state equation of gas is also considered.

### Computational Conditions

Both inside and outside surfaces of the gap are not slip wall or cold wall. The wall is set for an isothermal wall condition of 300K. The flow field is initialized by uniform flow which has the same inflow velocity. The initial air temperature is also 300K. The entrance, exit and the upper boundary are supposed to be pressure far fields boundary condition. The far field pressure equals to standard atmospheric pressure. In addition, The Mach numbers of free steam are 2, 3 and 4, respectively.

Constant-pressure specific heat *C_p_* = 1006.43J/kg·K and heat conduction coefficient equal to 0.0242W/m·K. The viscosity of flow can be determined by Sutherland Law

$$\frac{\mu }{\mu_{0}}={\left(\frac{T}{T_{0}}\right)}^{1.5}\left(\frac{T_{0}+T_{S}}{T+T_{S}}\right)$$

Where *µ* is the air viscosity corresponding to *T*, while *µ_0_* is the reference viscosity corresponding to a reference temperature *T_0_* = 231K, *T_S_* is the Sutherland constant 110K, and *µ*_0_ = 1.716 × 10^-5^.

### Computational Grids

**Fig 4:**
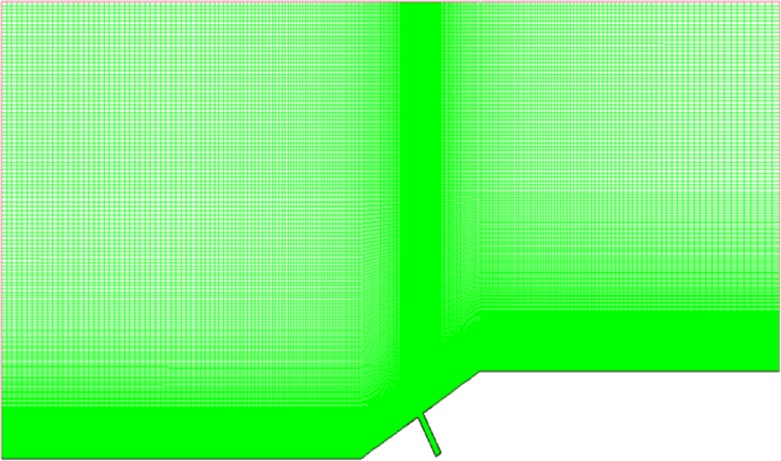
Computational grids (*α* = 30° and L/D = 1/6).

To get more accurate results, the grid is denser near the wall and in the gap. The sum of grids is approximately 230,000, and 70×270 grids in the gap. The distance between the nearest boundary layer grids and wall is 5×10^-6^m to guarantee the accuracy of calculation. Fig. 4 is the computational grid when *α* = 30° and L/D = 1/6. Moreover, there are 70×110 grids in the gap when L/D = 1/2, and 70×190 grids in the gap when L/D = 1/4. Grids outside the gap are as same as L/D = 1/6.

Note that dense triangle grid is employed in chamfer area and convex angle area. Different mesh densities were analyzed in a previous study and there was thin difference. The dense structural grid adopted in the paper is stable.

## Results

### Effect of Mach number on Gap Effect

**Fig 5:**
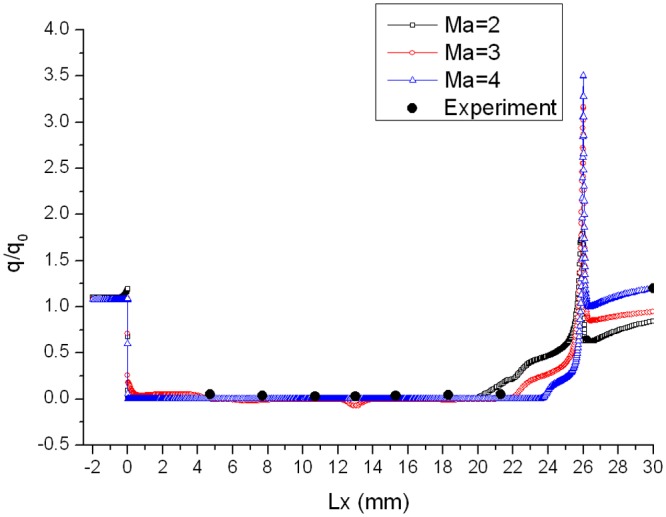
Distribution of heat flux ratio along the gap with L/D = 1/6 when *α* = 0°.

**Fig 6:**
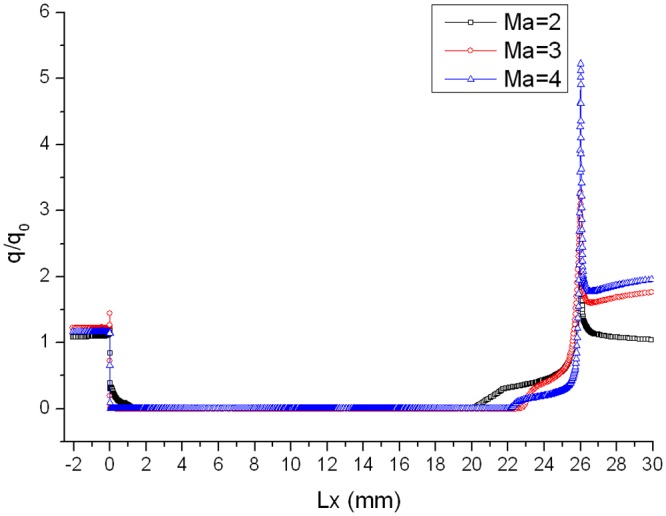
Distribution of heat flux ratio along the gap with L/D = 1/6 when *α* = 15°.

**Fig 7:**
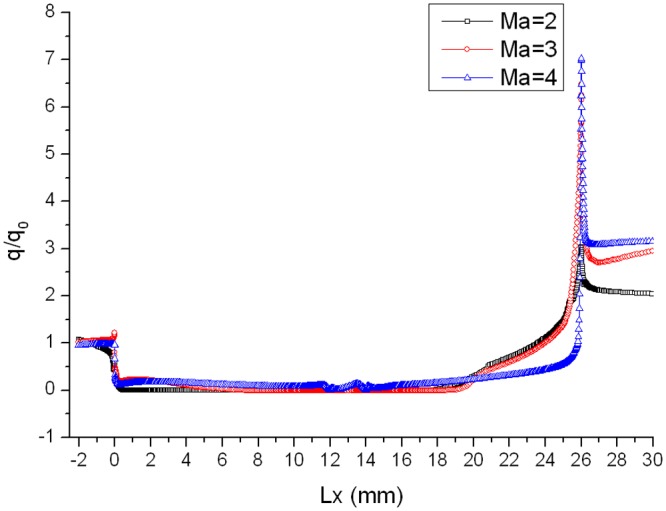
Distribution of heat flux ratio along the gap with L/D = 1/6 when *α* = 30°.

The ratio of gap heat flux *q* to the smooth plate heat flux *q*_0_ in the corresponding location is defined as the heat flux ratio. As are shown in Figs. 5, 6 and 7, curves of heat flux ratio in different conditions (L/D = 1/6, *α* = 0°,15°,30° and Ma = 2,3,4) can be gained by calculating the corresponding *q* and *q_0_*.

The heat flux ratio is basically U-shaped distribution along the surface of a gap. Owing to the impaction of high-speed flow onto windward of gap, heat flux ratio on windward is larger than that on leeward. The speed of flow at the bottom of the gap is almost 0, so the ratio is close to 0. Heat flux ratio in the gap decreases as Mach number increases, because the boundary layer tends to be thinner and air flowing into the gap decreases as Mach number increases.

In order to validate the reliability of the numerical simulation method mentioned in the paper, the result has been compared with one of supersonic velocity wind tunnel experiment, in which the data of the experiment came from reference [12]. The comparison of the heat flux ratio between numerical simulation and experiment (Ma = 5) when *α* = 0° is shown in Fig. 5. The simulation results match well with the experiment.

### Effect of Attack Angle on Gap Effect

**Fig 8:**
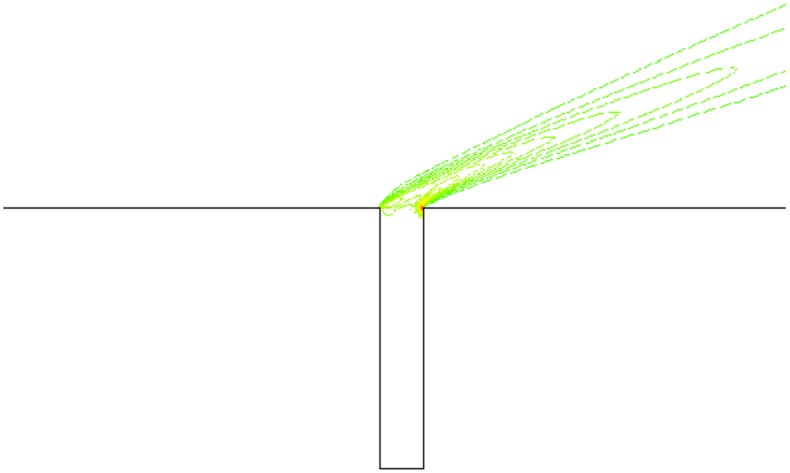
Isobar (*α* = 0°, Ma = 3 and L/D = 1/6).

**Fig 9:**
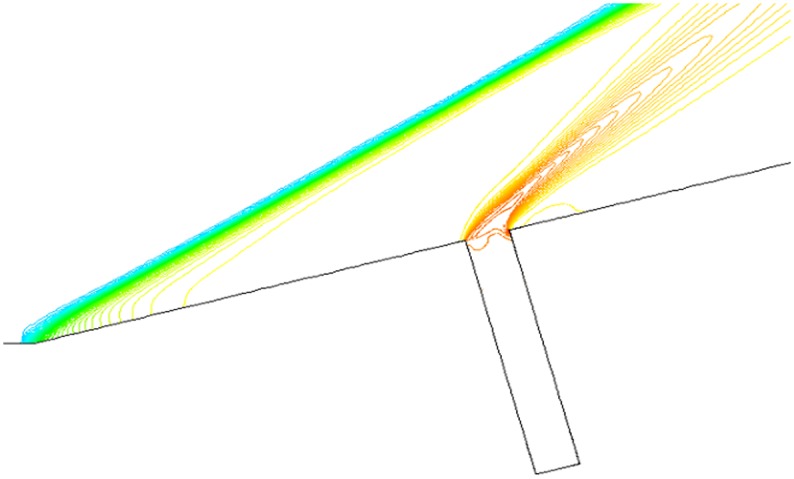
Isobar (*α* = 15°, Ma = 3 and L/D = 1/6).

**Fig 10:**
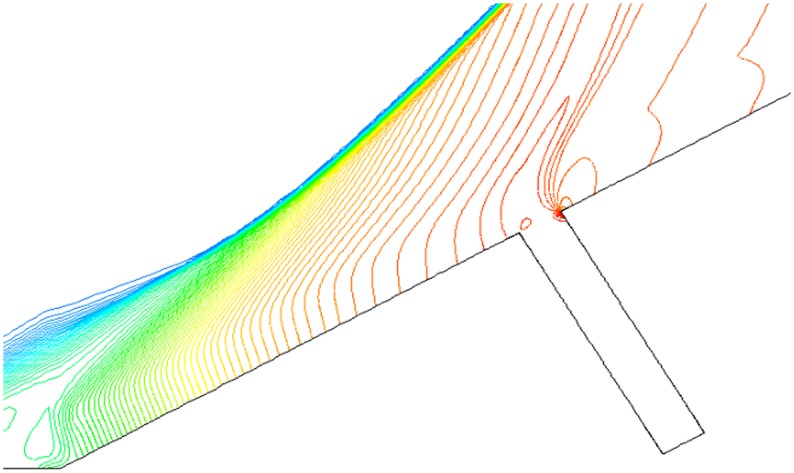
Isobar (α = 30°, Ma = 3 and L/D = 1/6).

Isobar under condition that *α* = 0°,15°,30° and Ma = 3 is shown in Figs. 8, 9 and 10. Pressure inside the gap is almost constant regardless of the attack angle, which shows that the flow speed must be low in the gap.

**Fig 11:**
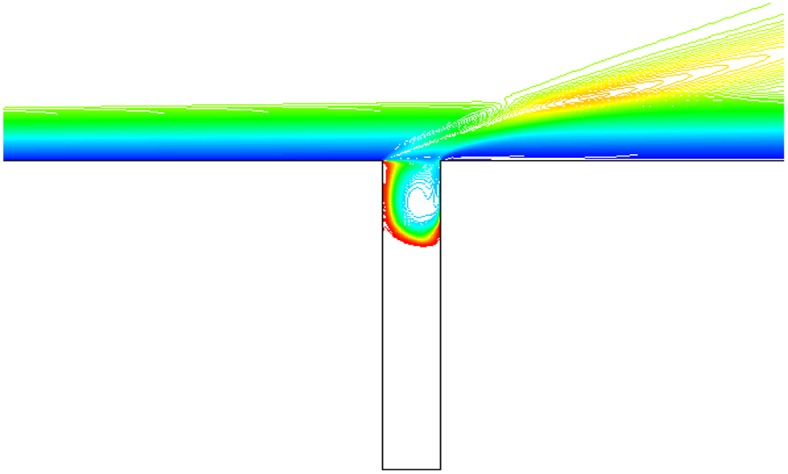
Isodensity pattern (*α* = 0°, Ma = 4 and L/D = 1/6).

**Fig 12:**
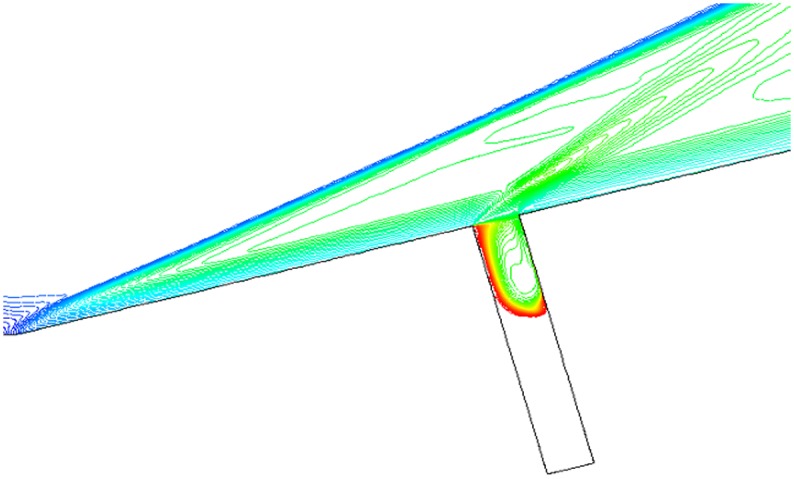
Isodensity pattern (*α* = 15°, Ma = 4 and L/D = 1/6).

**Fig 13:**
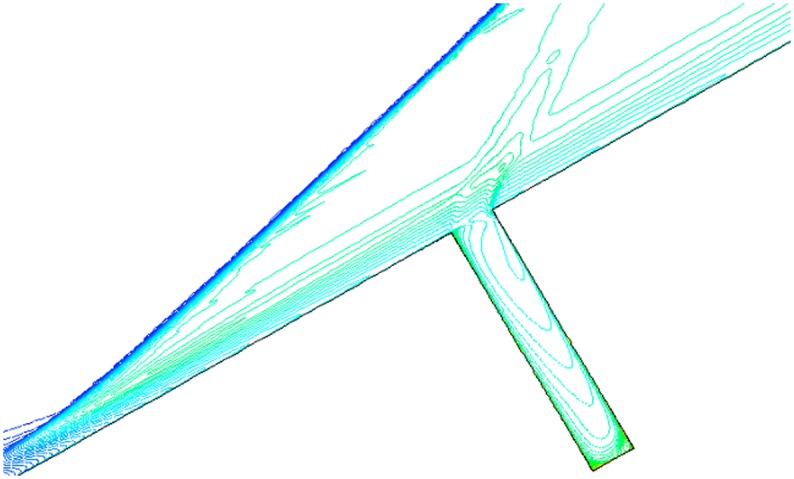
Isodensity pattern (*α* = 30°, Ma = 4 and L/D = 1/6).

Figs. 11, 12 and 13 present the isodensity lines under condition that *α* = 0°,15°,30° and Ma = 4. When α = 0°, the disturbance region of outflow only exists in the upper portion of gaps, which shows that the mass flow rate of outflow into gaps is little. The disturbance region is increasing, but is still a partial volume of the gap when *α* = 15°. However, when α = 30°, the disturbance region expands to the whole gaps.

**Fig 14:**
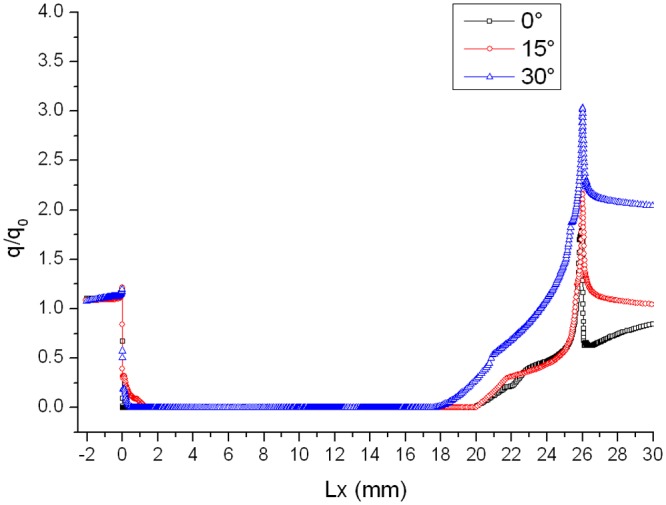
Distributions of heat flux ratio (Ma = 2 and L/D = 1/6).

**Fig 15:**
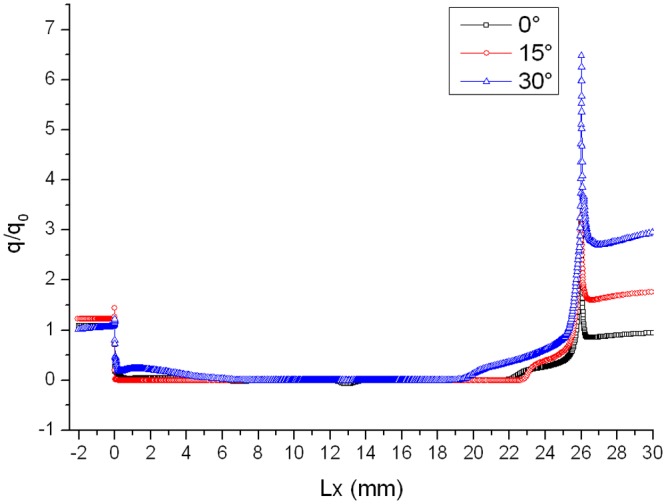
Distributions of heat flux ratio (Ma = 3 and L/D = 1/6).

**Fig 16:**
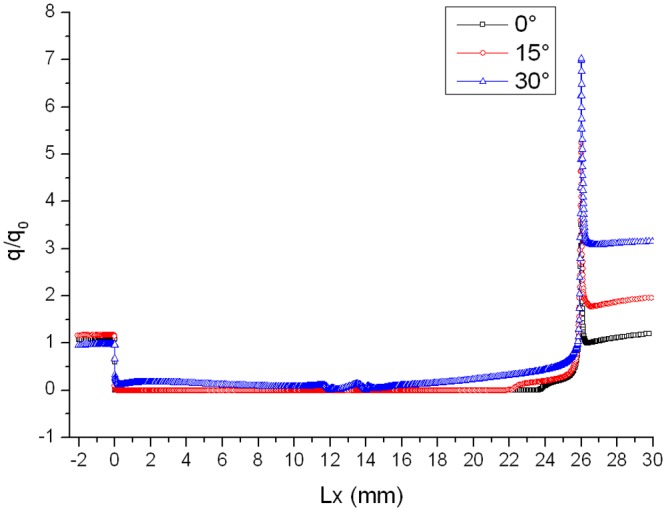
Distributions of heat flux ratio (Ma = 4 and L/D = 1/6).

Distributions of heat flux ratio in different conditions are reported in Figs. 14, 15 and 16. The heat flux at the bottom of the gap is almost zero and the curve of the heat flux ratio almost keeps horizontal as attack angle changes. However, the effect of attack angle on heat flux on the upper part of windward is obvious. The heat flux ratio increases in the upper area with the increase of attack angle, because the air flowing into the gap increases as attack angle increases.

### Effect of Width-to-depth Ratio on Gap Effect

**Fig 17:**
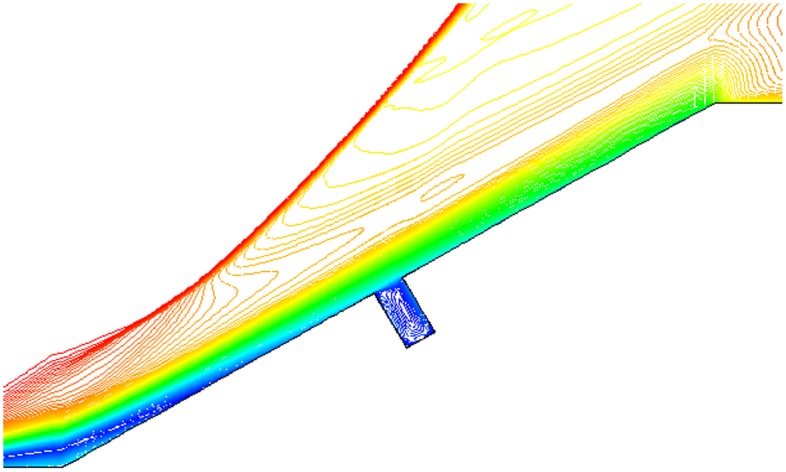
Isovelocity (L/D = 1/2, Ma = 3 and *α* = 30°).

**Fig 18:**
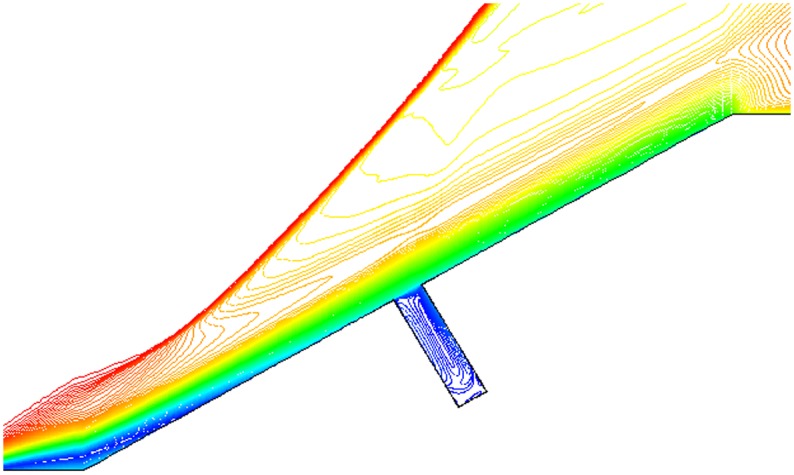
Isovelocity (L/D = 1/4, Ma = 3 and *α* = 30°).

**Fig 19:**
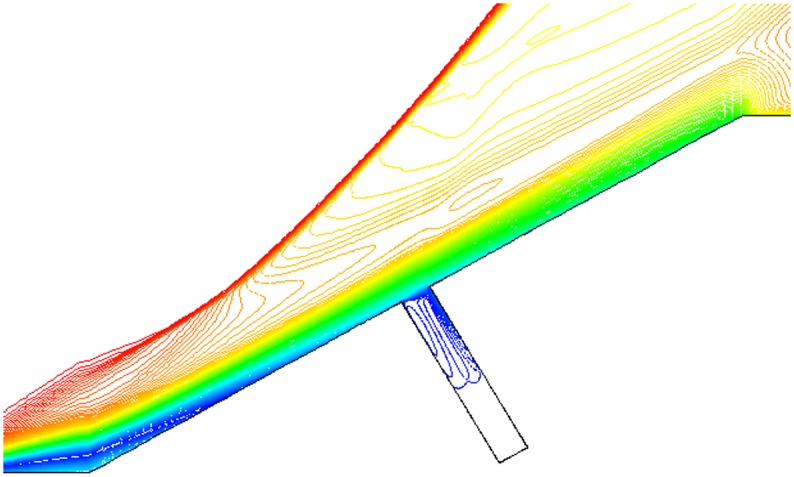
Isovelocity (L/D = 1/6, Ma = 3 and *α* = 30°).

Isovelocity under condition that L/D = 1/2,1/4,1/6, Ma = 3 and *α* = 30° is given in Figs. 17, 18 and 19. The isovelocity occupies the whole gap area in Fig. 17. In Fig. 18 it just occupies most part of the area and the speed of flow at the bottom part of the gap is quite low. In Fig. 19 the flow speed at most part of the gap keeps zero. As the depth of gap increases, the flow speed decreases.

**Fig 20:**
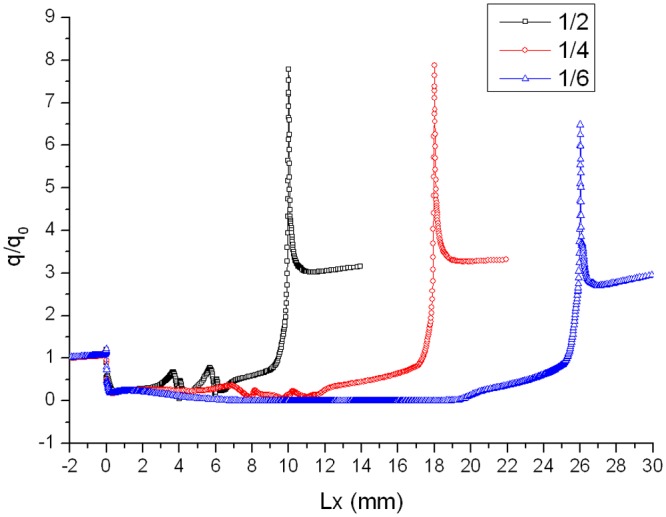
Distribution of heat flux ratio (Ma = 3 and *α* = 30°).

Distribution of heat flux ratio under condition that L/D = 1/2,1/4 or 1/6, Ma = 3 and *α* = 30° is shown in Fig. 20. It is obvious that the heat flux ratio at the bottom of the gap decreases as the width-to-depth ratio of the gap decreases. In Figs. 17 and 18 the flow speed at the bottom is not zero, but that in Fig. 19 is zero. The heat flux ratio is greater than zero at L/D = 1/2 or 1/4, but that keeps almost zero at L/D = 1/6.

### Gap Effect Coefficient

It is known that heat flux ratio always peaks at turning point of windward surface of the gap. The maximum of heat flux ratio is defined as the gap effect coefficient, which is used to represent degree of the gap effect in aerodynamic heating.

**Fig 21:**
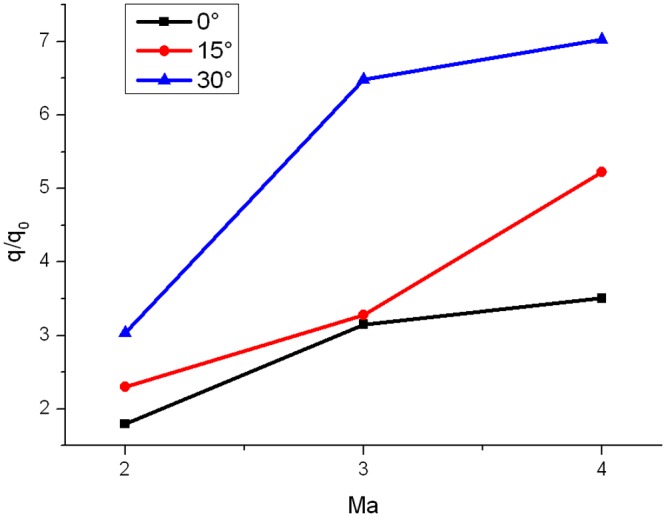
Distribution of gap effect coefficient (L/D = 1/6).

Distribution of gap effect coefficient under the condition that Ma = 2,3,4 and *α* = 0°,15°,30° is seen in Fig. 21. It can be seen from Fig. 21 that the gap effect coefficient increases with the increase of Mach number and attack angle.

**Fig 22:**
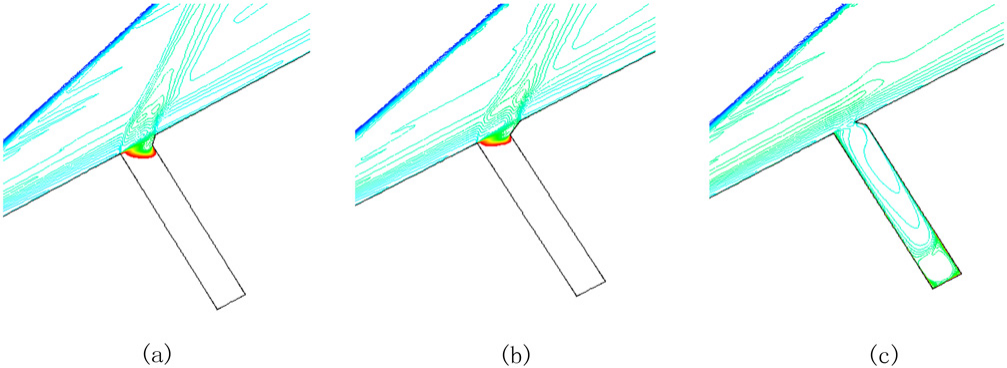
Isodensity pattern of model with chamfer and convex angle (L/D = 1/6). (a)chamfer(x = y = 0.5mm), (b)chamfer(x = 1mm, y = 0.5mm), (c)convex angle(x = y = 0.5mm).

In order to reduce the gap effect coefficient, chamfer and convex angle are set in the windward. Isodensity patterns (convex angle with x = y = 0.5mm, chamfer with x = y = 0.5mm or x = 1mm, y = 0.5mm) when Ma = 4 and *α* = 30° are described in Fig. 22(a), (b) and (c). Comparing Fig. 22(a) and (b) with Fig. 22(c), it is obvious that the effect of external flow with chamfer on gap only occurs in the open area of the gap. Part of high temperature air which could flow into gap through the chamfer, so the heat flux in gaps reduces effectively because of the chamfer.

Comparing Fig. 22(a) with Fig. 22(b), as the length x of the chamfer in slope direction increases from 0.5mm to 1mm and length y along the gap windward side is 0.5mm, the effect of small angle chamfer on incoming flow decreases. The isodensity lines of the model with convex angle are shown in Fig. 22(c). Owing to the existence of convex angle, external flow reaches the bottom of the gap.

**Fig 23:**
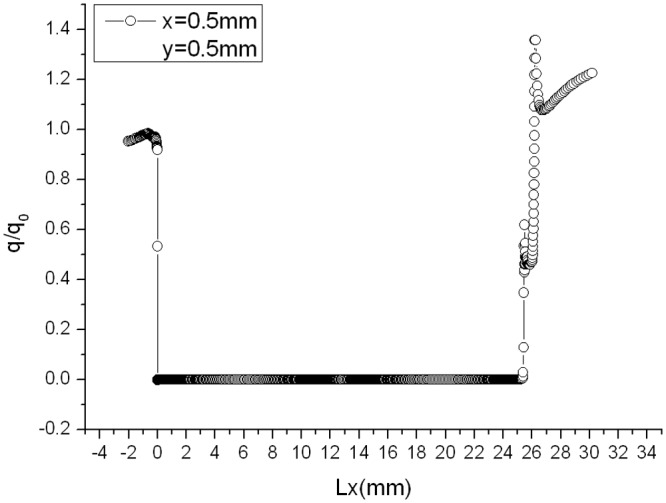
Distribution of gap heat flux ratio. (L/D = 1/6, Ma = 3, *α* = 30° and chamfer(x = y = 0.5mm))

**Fig 24:**
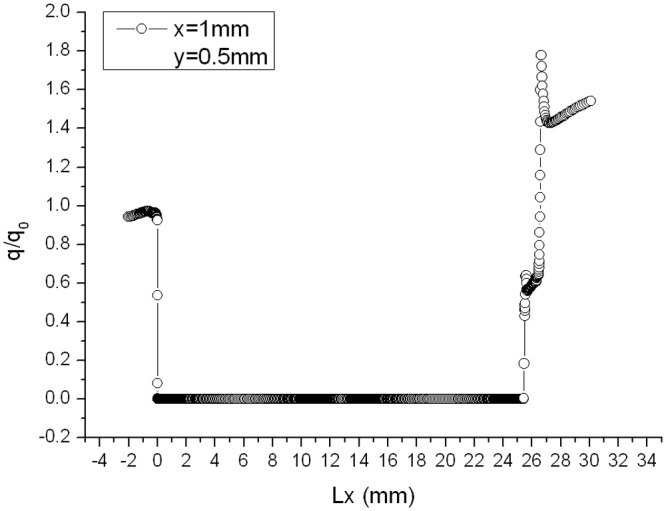
Distribution of gap heat flux ratio. (L/D = 1/6, Ma = 3, *α* = 30°and chamfer (x = 1mm, y = 0.5 mm))

Distributions of heat flux ratio of two models with chamfer under condition that Ma = 4 and *α* = 30° are shown in Fig. 23 and Fig. 24, respectively. It can be seen from Fig. 23 and Fig. 24 that the distribution of heat flux ratio along the surface of gap with chamfer is still U-shaped curve and except the opening area the heat flux ratio at most part of the gap almost equals to zero.

Compared with gap effect coefficient 7.028 in Fig. 7 (*α* = 30°, Ma = 4 without chamfer), it decreases to 1.358 when setting chamfer shown in Fig. 23, and to 1.78 in Fig. 24. Both gap effect coefficients are gained at the peak of the chamfer. Thus, setting chamfer in the windward can reduce the gap effect coefficient, which is a valid method for reducing the gap local aerodynamic heating environment.

**Fig 25:**
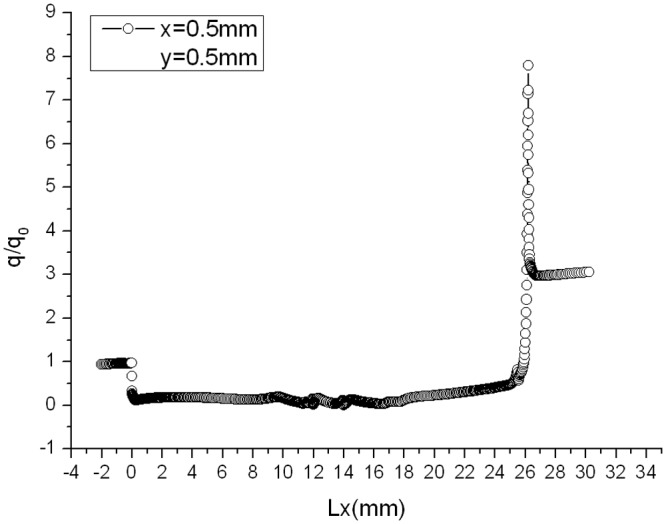
Distribution of gap heat flux ratio. (L/D = 1/6, Ma = 3, *α* = 30° and convex angle(x = y = 0.5 mm))

As shown in Fig. 25, gap effect coefficient goes up to 7.7945, compared with 7.028 without convex angle. A convex angle aggravates the aerodynamic heating in the gap. In order to prevent structure from failure, convex angle should be avoided in the design of engineering structure suffering from airflow at high temperature.

## Conclusions

Numerical simulation of two-dimensional flow around a gap is accomplished using the finite volume method. From the numerical results, it can be concluded as follows:

a) The heating ratio along the Lx coordinate in gaps shows ‘U’ curve approximately, the peak value appears at the corner of the windward surface of gaps because of the subsequent shock wave, and the gap effect depends not only on the attack angle, but also on the Mach number.

b) Chamfer in the windward corner can effectively reduce gap effect coefficient, whereas the convex angle would increase gap effect coefficient.
